# Job-loss and weight gain in British adults: Evidence from two longitudinal studies

**DOI:** 10.1016/j.socscimed.2015.08.052

**Published:** 2015-10

**Authors:** Pablo Monsivais, Adam Martin, Marc Suhrcke, Nita G. Forouhi, Nicholas J. Wareham

**Affiliations:** a Centre for Diet and Activity Research (CEDAR), MRC Epidemiology Unit, Institute of Metabolic Science, University of Cambridge, Cambridge, UK; b Health Economics Group, Norwich Medical School, University of East Anglia, Norwich, UK

**Keywords:** Unemployment, Obesity, Economic insecurity, Socioeconomic, Diet, Sleep-loss

## Abstract

Overweight and obesity have been associated with unemployment but less is known about changes in weight associated with changes in employment. We examined weight changes associated with job-loss, retirement and maintaining employment in two samples of working adults in the United Kingdom. This was a prospective study of 7201 adults in the European Prospective Investigation of Cancer (EPIC)-Norfolk study (aged 39–76 years) and 4539 adults in the British Household Panel Survey (BHPS) who were followed up over 43 months and 26 months, respectively. In both samples, changes in measured (EPIC) and self-reported (BHPS) weight were computed for each participant and assessed in relation to three employment transitions: maintaining paid employment, retirement and job-loss. Regression models adjusted for potential confounders. Further analyses evaluated the contribution of diet, physical activity and smoking to weight gain. In EPIC-Norfolk, weight change differed across the three employment transitions for women but not men. The mean (95% CI) annualised change in weight for women who became unemployed over the follow-up period was 0.70 (0.55, 0.85) kg/y while those who maintained employment gained 0.49 (0.43, 0.55) kg/y (P = 0.007). Accounting for changes in smoking, diet and physical activity did not substantially alter the difference in weight gain among groups. In BHPS, job-loss was associated with weight gain of 1.56 (0.89, 2.23) kg/y, while those who maintained employment 0.60 (0.53, 0.68) kg/y (P < 0.001). In both samples, weight changes associated with retirement were similar to those staying in work. In BHPS, job-loss was also associated with significant declines in self-reported well-being and increases in sleep-loss.

Two UK-based samples of working adults reveal strong associations between job-loss and excess weight gain. The mediating behaviours are so far unclear but psychosocial mechanisms and sleep-loss may contribute to the excess weight gain among individuals who become unemployed.

## Highlights

• Job-loss is associated with a more weight gain in working UK adults.
• The association between job-loss and weight may be stronger for women.
• Changes in smoking, diet and physical activity did not explain weight gain.
• Job-loss is associated with loss of sleep due to worry.
• Behavioural and psychosocial impacts of job-loss need further elucidation.

## 1 Introduction

There is both a scientific and policy interest in the relationship between employment and health in large part because unemployed adults have often been characterised as having unhealthy lifestyles (Bolton and Rodriguez, 2009; Schunck and Rogge, 2010), higher risk of chronic disease (Alavinia and Burdorf, 2008; Kessler et al., 1987) and higher risk of premature mortality (S. Montgomery et al., 2013; Morris et al., 1994). Unemployment has also been associated with unhealthy weight status, including overweight and obesity.

Population-level rates of unemployment have been positively associated with BMI and obesity in both cross-sectional (Akil and Ahmad, 2011; Slack et al., 2014) and longitudinal analyses (Latif, 2014). Individual-level studies have found a cross-sectional relationship between unemployment and underweight (S. M. Montgomery et al., 1998) and higher BMI among men (Schunck and Rogge, 2010). The associations for women have been more consistent, with unemployed women more likely to be overweight and obese than their employed counterparts (Kang et al., 2013; Rosmond and Bjorntorp, 1999; Sarlio-Lahteenkorva et al., 2006). Studies of individual-level employment history in samples of mixed sex found that more prolonged experience of unemployment was associated with higher BMI (Schunck and Rogge, 2010) and higher odds of obesity, particularly for women (Laitinen et al., 2002). However, no such associations were found in a sample of male construction workers (Leino-Arjas et al., 1999). Taken together, these studies provide evidence of an association between unemployment and higher weight, but there are still questions about whether unemployment leads to weight gain, and also how this association might differ by sex.

More robust prospective studies of job-loss and *change in weight* could help clarify the relationship between unemployment and excess weight. The few prospective studies found that employed persons who experienced job-loss gained more weight compared to those remaining in employment (Marcus, 2012; Morris et al., 1992). However, only one of these studies was UK-based (Morris et al., 1992) and both examined self-reported rather than measured weight. Furthermore, if job-loss is associated with weight gain, then there is also a need to examine concurrent behaviours that may contribute to weight gain. Changes in diet, physical activity and smoking have been associated with weight gain (Mozaffarian et al., 2011) and may also be linked to employment circumstances (Ali and Lindström, 2006; Dave and Kelly, 2012; Falba et al., 2005). Additionally, sleep disturbances are linked to obesity (Spiegel et al., 2009) and weight gain (Spaeth et al., 2013) and poor sleep quality has been linked to perceived job insecurity (Burgard and Ailshire, 2009), but little is known about the link between job-loss and sleep.

In two UK-based samples of employed adults, this study used a prospective design to examine the association between employment change and weight change among three groups: Those who maintained employment over the follow-up period, those who retired and those who became unemployed. Further analyses also explored whether changes in diet, physical activity and smoking affected the employment transition-weight gain relationship and examined changes in sleep and self-reported well-being during the employment transition.

## 2 Materials and methods

### 2.1 Study samples

**Fig. 1:**
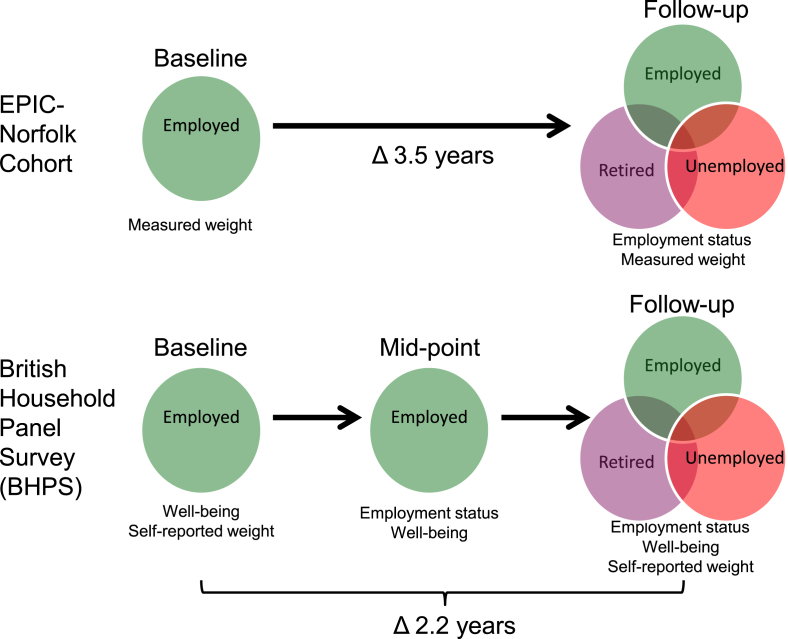
Schematic of timeline of measurements and reported employment data for the two samples of adults.

We used two population-based, longitudinal data sources which we describe in turn below. Because of the design and time frame for each of study samples, these two data sources allowed us to assess the generalisability of our findings by seeing whether associations of job-loss with weight change (our primary outcome) were consistent in different demographic, geographic and macroeconomic contexts. Moreover, the two data sources allowed us to explore a wider range of concurrent behaviours and secondary outcomes than if we had focused on only one data source. In both data sets, we focused on employed people to study weight changes in relation to three employment exposures: maintaining employment, retirement and job-loss. A schematic for the timeline for ascertainment of exposures and outcomes is illustrated in Fig. 1.

### 2.2 EPIC-Norfolk

The European Prospective Investigation of Cancer cohort study in Norfolk (EPIC) is a population-based study of dietary and lifestyle determinants of cancer and other chronic disease (Day et al., 1999). Recruitment was based on registers of general practices in the county. Participants were aged 39–76 at the time of entry (1993–97), where they completed questionnaires and dietary assessments and their weight and other measurements were recorded by trained research nurses. All volunteers gave written informed consent and the study was approved by the Norwich district ethics committee.

### 2.3 BHPS

The British Household Panel Survey (BHPS) was a multi-purpose, population-based longitudinal study of private households across Great Britain, conducted from 1991–92 to 2008–09 as an annual survey (‘wave’) of each adult member of a nationally-representative sample (Taylor et al., 2001). Only two waves (collected September 2004–May 2005 and September 2006–May 2007) included self-reported height and weight, and these formed the baseline and follow-up periods in the present study. In addition to these waves, data on employment status was also reported at an intermediate wave (‘mid-point’ in the present analysis, September 2005–May 2006). Conduct of the BHPS followed the ethical guidelines of the Social Research Association. No ethical approval was required for this secondary analysis of anonymised data.

### 2.4 Sample selection

In the EPIC study, of 25 639 adults recruited at baseline, restricting the sample to those who were employed at baseline and were weighed and provided data on key covariates, resulted in a sample of 12 210. Further restriction to include those for whom data on employment and health from an in-person follow-up were available resulted in an analytical sample of 7201. The BHPS was restricted to participants who reported details of their ‘current economic activity’ at follow-up and who had reported this as being ‘employed’ both at baseline and at midpoint. Excluding women who reported (at any of the three time points) as being pregnant or on maternity leave, students, people who reported being long-term sick and persons younger than 18 years of age and those lacking key covariates resulted in an analytical sample of 4539. The resulting data sets represented a complete-case analysis. A depiction of the restriction of both samples is presented in Supplementary Fig. S1. To examine the potential for sample bias resulting from this selection process, we made a quantitative comparison of the demographic, socioeconomic and other characteristics of the analytical and full samples of employed adults in both data sets. The comparison, shown in Supplementary Table S1, indicated that both analytical samples were similar to the full samples in most respects, with the analytical sample having slightly more women in the EPIC sample but fewer women in the BHPS sample. In both samples, restriction resulted in slightly fewer smokers and people in moderate or poor health.

### 2.5 Exposure: transitions in employment status

Classification of employment transitions in the EPIC and BHPS data was based on the reported employment status at follow-up. In EPIC, those classified as remaining employed had stated they were working at follow-up (n = 5144, 71%). Those who were classified as retired had indicated they were retired and not working (n = 1327, 18%) as well as those who defined themselves as retired but also reported working (n = 226, 3%). The ‘lost job’ category combined all those who were not working at follow-up and also defined themselves as unemployed (n = 154, 2%), unemployed and retired (n = 130, 2%) and persons who were not employed or retired but did not provide any other reason for being out of work (n = 195, 3%). We reasoned that this inclusive approach to defining the ‘lost job’ category was important for older adults, who nearing the end of their working life, when confronting the loss of a job might have ambiguous feelings about their status and perhaps elect to regard themselves as partly retired (e.g., both unemployed and retired) or otherwise not identify as unemployed if they were taking on unpaid domestic or caring roles for spouses or other family members. A fourth group ‘mixed classification’ included individuals who identified themselves as simultaneously employed, retired and unemployed (n = 25).

When querying employment situation at each time point, the BHPS questionnaire provided response options for being employed, self-employed, retired, unemployed as well as such activities as taking a role as family carer, becoming sick or disabled and participation in a government training scheme, among other options. Thus, the BHPS, employment status was unambiguous and participants were classified as ‘remained employed’ if they had reported being ‘employed’ at all three waves. Participants were classified as ‘retired’ (n = 75, <2%) or ‘lost job’ (n = 53, 1%) if they stated ‘retired’ or ‘unemployed’ as their current economic activity at follow-up, respectively.

### 2.6 Primary outcome: weight change

The outcome was change in body weight between baseline and follow-up, which was based on a variable computed for each participant. Because the average length of follow-up differed between the two data sources, an annualised weight change variable was computed as follows. Weight (kg) at baseline was subtracted from weight at follow-up and divided by the length of the follow-up period (in days, which varied among participants in both data sets) and multiplied by 365. The variable was kg/y.

### 2.7 Secondary outcomes: well-being and sleep-loss

An overall measure of psychological well-being and a specific indicator of sleep-loss were secondary outcomes in analysis of the BHPS (similar data were not available in EPIC). Both of these outcomes were obtained from a 12-item General Health Questionnaire (GHQ12), a widely-used and validated instrument for assessing psychological wellbeing in patient populations and in the UK general population (Goldberg and Williams, 1988; Hardy et al., 1999). At all three time points examined in this study, participants in BHPS were administered the GHQ12, to capture participants' ratings of the twelve specific psychological symptoms on a four-point scale (0–3) (Bowling, 2004; Goldberg and Williams, 1988). Responses were used to derive a continuous indicator of overall psychological wellbeing on a 36-point scale. In addition to the overall indicator, we were also interested in responses to the GHQ12 question on sleep-loss. The question was: ‘*Have you recently lost sleep over worry?*’ with responses ranging from ‘*not at all*’ (0) to ‘*much more than usual*’ (3).

### 2.8 Covariates

In main analyses with both data sets, covariates were selected *a priori* and included basic demographic and socioeconomic characteristics reported at baseline: age, sex, and educational attainment, smoking status and measured body weight. In the EPIC data set, we also examined three health behaviours that might contribute to weight change: smoking status, dietary intake and physical activity, which were assessed at both baseline and follow-up. Dietary intake was assessed with a semi-quantitative food frequency questionnaire (Bingham et al., 2001) at both times, which allowed the derivation of estimated energy intake. Occupational and leisure-time physical activity was assessed with a short questionnaire at baseline (Wareham et al., 2003) and a more comprehensive questionnaire at follow-up (Wareham et al., 2002). Physical activity was a four-category, ordinal variable that has been previously validated against objectively-measured physical activity (Wareham et al., 2003). The categories were ‘inactive’, ‘moderately inactive’, moderately-active’, and ‘active’. Change variables for each of the three behaviours were constructed based on any difference between baseline and follow-up. These were: change in smoking status (four categories: ‘remained non-smoker’, ‘quit smoking’, ‘started smoking’ and ‘remained a smoker’), change in total dietary energy intake (continuous MJ/d), and physical activity level (three categories: decline in physical activity, no change in physical activity, and increase in physical activity).

### 2.9 Analytic approach

In both EPIC and BHPS, analyses examined the association between employment transition and weight change using multiple linear regression models to adjust for covariates. These models provided estimates of mean weight change for each of the three employment groups, adjusted for sex, age, smoking status at baseline, educational attainment and baseline body weight. In EPIC, the ‘mixed classification’ employment transition group was included in all models but estimates were not reported due to the small sample size (n = 25). Analysis of EPIC also permitted sex-stratified models to produce estimates for men and women separately. Significant differences across groups were assessed by making pair-wise comparisons between the group maintaining employment (the reference group) against the other two groups. In further analyses of the EPIC data set, we adjusted for changes in three behaviours that might have explained the association between employment transition and weight change. These models were based on a further restricted sample in which data on smoking, diet and physical activity was available at both time points (n = 5401). The base model did not account for any health behaviours and each additional model progressively adjusted for change in smoking, diet, and physical activity.

In the BHPS, changes in psychological well-being and sleep-loss due to worry were evaluated using multiple linear regression for a continuous outcome (well-being) and multivariable logistic regression for dichotomous outcome (sleep-loss). The four response levels of sleep-loss were dichotomised with the two lowest categories being assigned the value zero and the higher two categories (i.e., experiencing more sleep-loss) being assigned one.

### 2.10 Sensitivity analyses

The two data sets permitted different sensitivity analyses. In the BHPS, we conducted a number of analyses further restricting the analytic sample, so that the impact of potential sources of bias could be explored. These included the exclusion of individuals with irregular employment histories (defined as not having been in employment in two study waves prior to baseline) and those who reported being employed on temporary (rather than permanent) basis as well. A further analysis attempted to address the possibility of health selection (Bartley and Ferrie, 2001) by excluding those who experienced a substantial deterioration in their health status between baseline and follow-up (which we defined as a worsening in self-rated health status from Excellent to Fair or worse, Good to Poor or worse, or Fair to Very Poor).

In both BHPS and the EPIC cohort, we examined the impact of additionally adjusting for occupational social class of the participant and baseline marital status, BMI (instead of baseline weight), and height at baseline. Occupational social class, which was recorded in six categories, was reduced to a binary variable (non-manual versus manual). Marital status, recorded in five categories, was reduced to a binary variable (married versus not married). Baseline BMI, in kg/m^2^ and height, in cm, were used as a continuous terms.

All analyses were conducted using Stata version 12.1 for PC (College Station, USA) and SPSS Statistics 19.0 for PC (IBM, Chicago, USA).

## 3 Results

### 3.1 Characteristics of the two samples

The EPIC-Norfolk sample was older, had a higher percentage of women and married people, and included a lower percentage smokers compared to the BHPS sample. The EPIC-Norfolk sample also had a higher percentage of people who reported being in good/excellent general health compared to the BHPS sample. The EPIC sample had a lower BMI than the BHPS sample 25.9 versus 26.8 kg/m^2^ and unadjusted annualised weight gain over the follow-up period was also lower in EPIC than in BHPS (0.49 versus 0.61 kg/y). Detailed demographics of both samples overall and by gender are provided in Supplementary Tables S2 and S3.

### 3.2 Characteristics by employment transition

The two samples showed different sociodemographic patterning among the three employment groups (Table 1). Of the 7201 adults in the EPIC sample, 5144 (71%) stayed in employment at the follow-up, 1553 (22%) entered retirement and 479 (7%) had become unemployed or otherwise were no longer working. Most sociodemographic characteristics were similar across the three groups and baseline health status and weight were also similar. Only the proportion of women and age appeared to vary systematically across groups. In the BHPS, a much smaller percentage either entered retirement (2%) or became unemployed (1%). Unlike in the EPIC sample, demographic, socioeconomic and health characteristics showed more pronounced differences across the three groups. In particular, those who became unemployed were less likely to be married at baseline and had lower educational attainment compared to those who either stayed employed or retired. This group was also more likely to smoke and less likely report their health status at baseline as ‘good’ or ‘excellent’ compared to either those who stayed in employment or who retired.

### 3.3 Weight change by employment transition

Analyses of the EPIC cohort identified significant differences in measured weight gain among groups in the interval between baseline and follow-up. Table 2 shows that unadjusted weight change was greatest in those who lost their job during the interval and significantly different from the other two groups. After adjusting for age, sex and a number of other potential confounders, the results were similar. While those who were still employed at follow-up gained an average (95% CI) of 0.51 (0.47, 0.55) kg/y, those who had become unemployed gained 0.65 (0.54, 0.76) kg/y (P = 0.014). Weight gain for those who entered retirement during the interval was equivalent to those who had remained employed: 0.49 (0.42, 0.57) kg/y. In the BHPS, a similar pattern emerged among the three groups in the amount of weight gain over the interval. Adjustment for potential confounders of weight gain had little effect on the estimated means. Among those who lost their jobs, estimated weight gain was 1.56 (0.89, 2.23) kg/y while those who stayed in employment gained 0.60 (0.53, 0.68) kg/y (P < 0.001).

### 3.4 Weight change by employment transition and sex

**Fig. 2:**
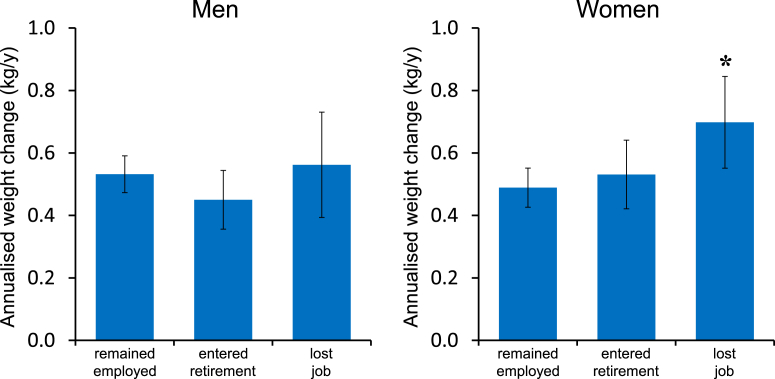
Estimated mean and 95% CIs of annualised weight change between baseline and follow-up for each group defined by employment transition for men (left) and women (right). Estimates are adjusted for age, baseline smoking status, baseline weight, and educational attainment. Asterisk indicates significantly different from employed group, P = 0.007. Data from the EPIC-Norfolk sample (n = 7201).

Men and women differed in the extent to which weight gain was associated with job-loss. Multiple linear regression models were used to produce sex-specific estimates of weight change in relation to employment transitions from the EPIC-Norfolk cohort indicated that the weight gain associated with job-loss was disproportionately experienced by women in the sample (Fig. 2). While there were no significant differences in weight gain associated with changing employment among men (left panel), differences were observed across the three groups among women (right panel). While women who remained employed gained an average of 0.49 (0.43, 0.55) kg/y, those who experienced job-loss gained an average of 0.70 (0.55, 0.85) kg/y over the follow-up period (P = 0.007). Women who entered retirement gained a similar amount of weight as those who stayed in work, gaining 0.53 (0.42, 0.64) kg/y over the period.

### 3.5 Assessing the contribution of health behaviours to differences in weight gain

Sex-specific models of weight gain from the EPIC-Norfolk cohort were used to explore the potential contribution of smoking, diet, and physical activity to the observed differences in weight gain among men and women. Accounting for changes in these behaviours did not affect estimates of weight change across the three employment groups (Table 3). Not accounting for any of these three health behaviours (Model A) produced similar results to those reported in Fig. 2, which had adjusted for smoking status at baseline. Adjusting for changes in smoking (Model B) and then further adjusting for changes in dietary energy intake (Model C) and changes in physical activity level (Model D) did not affect the estimated weight changes in the three groups.

### 3.6 Assessing changes in wellbeing and loss of sleep in relation to job-loss

**Fig. 3:**
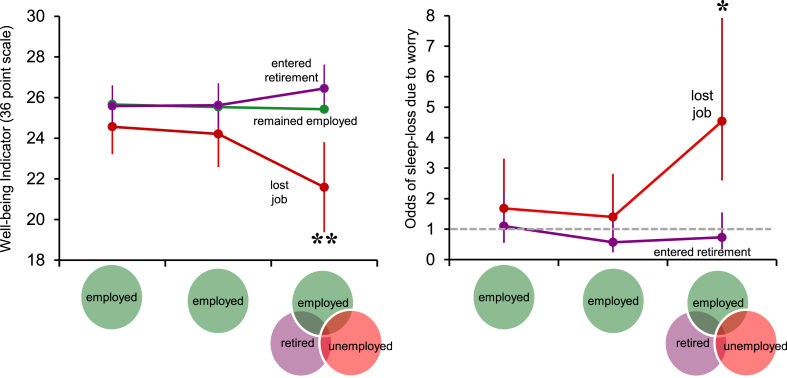
Left panel, mean and 95% CIs of scores for overall psychological well-being, based on the General Health Questionnaire 12 (36-point scale) at baseline, midpoint and follow-up for each group defined by employment transition. Right panel, odds ratios and 95% CIs for experiencing sleep-loss due to worry for those who entered retirement and became unemployed. Reference group for each time point are those who maintained employment. Estimates are adjusted for age, sex, educational attainment and duration of follow-up. Double asterisks indicate significantly different from employed group, P < 0.005; Single asterisk indicates difference from employed group P < 0.01. Data from the British Household Panel Survey (n = 4539).

In BHPS, changes in overall psychological wellbeing, based on the GHQ12, are shown in the left panel of Fig. 3. At baseline and mid-point, when participants in all three groups were still in paid employment, no statistically significant differences were observed in well-being. At follow-up, those in the job-loss group reported a significant decline in overall psychological wellbeing of approximately 3 points (P < 0.005 comparing to those remaining employed) while those who either stayed employed or retired reported no such change. Sleep-loss due to worry, one of the 12 GHQ components, also showed differences across groups, but these differences only became apparent at follow-up for the job-loss group. The right panel of Fig. 3 shows the odds of sleep-loss for participants who would eventually go into retirement or become unemployed at follow-up, relative to those who maintained employment throughout. At follow-up, those who experienced job-loss showed a sharp and significant rise in the odds of sleep-loss, with odds rising to 4.5 relative to those maintaining employment (P < 0.01). There were no significant differences for the group retiring at follow-up. Among the other components of the GHQ12, feelings of unhappiness, depression, being worthless, losing confidence and unable to enjoy normal day-to-day activities all showed significant rises at follow-up for those who became unemployed (data not shown).

### 3.7 Results of sensitivity analyses

Additional analyses in BHPS excluded participants who had weak or transient attachment to the labour market (Supplementary Table S4). These analyses excluded persons holding temporary jobs at baseline (Model 2), persons who were not employed at both baseline and the preceding wave (Model 3) and those who were not employed at baseline and the two consecutive waves preceding baseline (Model 4). These analyses also examined the impact of excluding those who were in poor health at baseline or experienced substantial deterioration in health (Model 5). Across all models, the impact of job-loss remained statistically significant with effect sizes (differences between job-loss and employed groups) ranging from 0.80 kg/y (Model 3) to 1.05 kg/y (Model 2).

Analyses in both BHPS and EPIC-Norfolk indicated that the magnitude or significance of estimated weight gain among the three employment groups was not affected by adjustment for occupational social class, marital status or by adjusting for baseline BMI or height (data not presented).

## 4 Discussion

We found that working adults who experience job-loss (i.e., become unemployed) gain more weight than those who either stay employed or enter retirement. The analyses, conducted using two distinct, high-quality UK data sources (a population-based cohort and a national panel survey), further suggest that the associations might be stronger in women. Moreover, while any contribution of smoking, diet and physical activity to excess weight gain could not be detected, job-loss was associated with a decline in overall psychological well-being and increased odds of experiencing sleep-loss due to worry. The present study complements and expands on existing literature by examining longitudinal associations between employment changes and weight and by examining concurrent health behaviours.

Our findings may also have implication for understanding socioeconomic inequalities in obesity. Low socioeconomic status been consistently linked to higher weight and weight gain (*Adult obesity and socioeconomic status data factsheet*, 2014; Ball and Crawford, 2005; McLaren, 2007), and notably, many of these studies find that associations between SES and weight are more consistent among women than men. The EPIC-Norfolk results suggested that weight gain associated with job-loss was greater in women and previous studies have reported that the experience of job insecurity (Ferrie et al., 1998) unemployment (Laitinen et al., 2002) and job-loss (Marcus, 2012) is more strongly associated with adiposity outcomes in women than in men. While our analyses attempted to control for socioeconomic status, the adverse effects of job-loss may amplify existing socioeconomic inequalities in weight, since job-loss and unemployment are experienced more by those of lower socioeconomic classes (Davies, 2014).

If job-loss causes weight gain, the underlying mechanisms are still to be determined. Unemployment's adverse effects on health are thought to be mediated both by material pathways, including lost income and reduced standard of living, as well as via psychosocial pathways, including anxiety, depression, lower self-esteem and reduced social integration (Marmot, 2010; Thomas et al., 2007). Lost income and any accompanying financial hardship may be relevant for weight gain. The experience of financial hardship and economic insecurity have been shown to be associated with higher weight (Lynch et al., 1997), greater odds of obesity (Conklin et al., 2013; Laaksonen et al., 2004) and more weight gain (Conklin et al., 2014; Loman et al., 2013; Smith et al., 2009). Economic insecurity has also been associated with food choices that promote weight gain (Smith, 2012) and studies have found that the purchase and consumption of more energy-dense foods rises during times of economic recession (Griffith et al., 2013) and in areas with rising rates of unemployment (Dave and Kelly, 2012).

### 4.1 Identifying behavioural contributors to weight gain associated with job-loss

In our analysis of EPIC-Norfolk, we did not detect a contribution of diet to weight gain. Our failure to detect a contribution of diet may have been due to the limitations of food frequency questionnaires (Kristal et al., 2005; Willett, 2013), which we used for dietary assessment. It may also be the case that the relevant characteristics of diet related to weight gain may have been its composition, which we did not examine in this study. In our analyses, we characterised diet only in terms of total energy intake and changes in energy intake from baseline to follow-up.

Physical activity and smoking were also examined in this study because these behaviours have been linked to both employment and weight. For example, some studies have found lower levels of leisure-time physical activity among unemployed groups (Ali and Lindström, 2006), and leaving employment might have led to lower levels of physical activity for individuals who worked in manual occupations (Barnett et al., 2013). Smoking and changes in smoking status have profound influence on weight and weight gain (Filozof et al., 2004; Williamson et al., 1991) and smoking is more prevalent among unemployed groups (Cook et al., 1982; Lee et al., 1991). Some studies have reported that those who experienced job-loss or retired involuntarily were more likely to take up or intensify their smoking (Falba et al., 2005; Henkens et al., 2008). However in the analysis of EPIC data, estimates of weight gain were not affected by the adjustment for either physical activity or smoking status. This leads us to suggest that these behaviours, as defined here, were not contributing to differences in weight gain between groups.

Sleep-loss was examined as part of an overall measure of well-being. Short sleep duration and poor sleep quality have been linked to obesity (Cappuccio et al., 2008; Spiegel et al., 2009) and weight gain (Markwald et al., 2013; Spaeth et al., 2013). We found that job-loss in the BHPS coincided with increased odds of sleep-loss due to worry and a deterioration of overall psychosocial well-being. The results align with previous studies, which found that unemployment, job-loss and job insecurity were associated with anxiety (Kessler et al., 1988), depression (Burgard et al., 2009), overall declines in psychological well-being (Flint et al., 2013; Thomas et al., 2005) and difficulty sleeping (Burgard and Ailshire, 2009).

### 4.2 Methodological considerations and limitations

Our use of two samples was an important feature of this study that requires some discussion. Weight gain associated with job-loss was lower in EPIC-Norfolk than in BHPS and this might have been due to a number of factors. In particular, EPIC is composed of older adults who generally gain weight at a lower rate than younger adults (Sheehan et al., 2003) and this was confirmed in the overall rates of weight gain in the two samples. Furthermore, the definition of job-loss differed between the two samples. The job-loss group in BHPS was limited to those who declared themselves unemployed at follow-up. In EPIC-Norfolk, job-loss group was more inclusive (see Methods) and may have classified people as ‘job-losers’ who, while not employed, were nevertheless engaged in activities that provide structure and social meaning, which are thought to be integral to the beneficial effects of paid employment (Bartley, 1994).

There were several limitations in this study. First, our classification of any changes in individuals' employment status was based on self-reported employment status at two (EPIC) or three (BHPS) discrete time points. Thus, we did not know exactly when job-loss occurred between time points. Second, we could not ascertain the reason for unemployment, which may have introduced bias into the analysis, if for example, an individual developed a disability or illness that led to both job termination and weight gain (Bartley and Ferrie, 2001; Deb et al., 2011; Marcus, 2012). We attempted to address this concern in the BHPS in sensitivity analyses that excluded individuals who had experienced ill health or substantial deterioration in health, and these analyses showed similar results. Third, in the EPIC cohort, we also had limited information about individuals' employment histories or stability of employment prior to our study baseline. Some of those who we classified as employed at baseline may have been only weakly or transiently attached to the labour market and hence might have been misclassified. However, this limitation was partly addressed in the BHPS, which allowed us to restrict that sample to adults who had stable histories of employment prior to the baseline used for analysis. Different levels of restriction based on labour market history did not substantially affect the results (See sensitivity analyses in Supplementary Table S4). Another limitation was the limited duration of follow-up. The interval between baseline and follow-up was on average 3.5 years (EPIC) and 2.2 years (BHPS). Longer-term outcomes would have been valuable but these would have been difficult to interpret without continued ascertainment of employment status.

These limitations were balanced by a number of strengths including the use of individual-level longitudinal data to study the impact on weight change of becoming unemployed. Much of the literature on employment and adiposity use area-level data, cross sectional data and/or only self-reported weight. By finding similar results in a cohort and national panel data with strikingly different sociodemographic, geographic and time context, the present study indicates that the associations between job-loss and weight gain are generalisable and robust against some of the sources of bias described above. Further, the combination of these two data sets allowed us to explore several potential behavioural mechanisms for the observed differences in weight gain.

### 4.3 Conclusions

Safe and secure employment is a recognised determinant of population health (Davies, 2014; Marmot, 2010) and our findings revealed that becoming unemployed was associated with significantly greater weight gain in two samples of working adults in the UK. The relatively short intervals over which weight gain became apparent suggests that longer periods of unemployment or insecure employment may have substantial cumulative effects on population weight status and health. More research is needed to understand how job-loss leads to weight gain and ill health and the contribution of health behaviours. The substantial declines in overall psychosocial well-being and increased sleep-loss associated with job-loss. The implication is that, at least in the short-term, health impacts of job-loss might be mainly mediated through psychosocial pathways and sleep-loss.

## Figures and Tables

**Table 1:** Baseline characteristics of the two population samples overall and by employment transition category. Data from the EPIC-Norfolk sample (n = 7201) and the British Household Panel Survey (n = 4539).

Sample	Employment transition^d^	n	Women (%)	Age^a^ (mean y)	Married^a^ (%)	Education (A-level or degree) (%)	Higher occupational social class^b^ (%)	Current smoker^a^ (%)	Health status^a^ (% good or excellent)	Weight^c^ (mean kg)
EPIC-Norfolk	Full analytic sample	7201	52.5	53.4	86.4	61.6	46.2	10.4	88.0	73.5
Remained employed	5144	52.3	51.3	86.9	63.2	46.1	10.7	88.9	73.3
Entered retirement	1553	49.6	60.0	85.5	57.1	46.3	9.0	86.8	74.6
Lost job^e^	479	64.0	54.4	81.9	58.2	44.5	13.3	81.4	72.7

BHPS	Full analytic sample	4539	36.7	41.8	61.8	50.1	42.3	23.5	80.2	80.0
Remained employed	4411	36.7	41.5	61.8	50.5	42.3	23.4	80.5	80.0
Entered retirement	75	38.7	59.8	77.3	40.0	50.0	14.7	80.0	80.9
Lost job	53	30.2	39.3	34.0	30.2	26.9	41.5	62.3	84.6

a Reported at baseline.

b Professional or Managerial and technical professions.

c Measured at baseline in EPIC-Norfolk, self-reported weight in BHPS.

d In EPIC-Norfolk, a fourth employment transition group of mixed classification (n = 25) is not displayed.

e This group includes individuals who left paid employment after baseline and either declared themselves unemployed at follow-up (n = 284) or did not indicate reason for being out of work and were not retired (n = 195).

**Table 2:** Changes in annualised weight per year in two population samples, by employment transition category. Weight changes are estimated in multiple linear regression models with no covariates and with progressive adjustment for potential confounders. Data from the EPIC-Norfolk sample (n = 7201) and the British Household Panel Survey (n = 4539). EPIC results based on measured weight and BHPS results based on self-reported weight.

Sample	Employment transition^a^	n	Annualised mean change and 95% CI in body weight (kg/year) over follow-up period				
unadjusted	age and sex adjusted	+ smoking status at baseline	+ weight at baseline	+ educational attainment
EPIC-Norfolk	Remained employed	5144	0.52 (0.48, 0.55)	0.49 (0.45, 0.52)	0.52 (0.48, 0.56)	0.52 (0.48, 0.56)	0.51 (0.47, 0.55)
Entered retirement	1553	0.38 (0.32, 0.44)	0.47 (0.40, 0.54)	0.50 (0.43, 0.57)	0.50 (0.43, 0.57)	0.49 (0.42, 0.57)
Lost job^b^	479	0.62 (0.51, 0.72)	0.63 (0.52, 0.74)	0.66 (0.55, 0.77)	0.66 (0.55, 0.77)	0.65 (0.54, 0.76)

BHPS	Remained employed	4411	0.61 (0.53, 0.68)	0.60 (0.53, 0.68)	0.60 (0.53, 0.68)	0.60 (0.53, 0.67)	0.60 (0.53, 0.68)
Entered retirement	75	0.38 (−0.18, 0.95)	0.66 (0.08, 1.24)	0.67 (0.09, 1.25)	0.67 (0.09, 1.24)	0.67 (0.09, 1.24)
Lost job	53	1.54 (0.86, 2.21)	1.51 (0.83, 2.19)	1.48 (0.80, 2.16)	1.58 (0.91, 2.25)	1.56 (0.89, 2.23)

a In EPIC-Norfolk, a fourth employment transition group of mixed classification (n = 25) was included in all models but not displayed.

b This group includes individuals who left paid employment after baseline and either declared themselves unemployed at follow-up (n = 284) or did not indicate reason for being out of work and were not retired (n = 195).

**Table 3:** Mean (95% CI) annualised weight change in kg/year by employment transition category before and after accounting for health behaviours. Estimates are produced in multiple linear regression models that progressively adjusted for behavioural factors. Data from the EPIC-Norfolk sample and analyses further restricted to women with valid dietary data, and physical activity estimates at both time points (n = 5401).

	Employment transition^e^	Annualised mean change and 95% CI in body weight (kg/year) over follow-up period			
*Model A*^a^:	*Model B: A with change in smoking status*^b^	*Model C: B with change in dietary energy intake*^c^	*Model D: C with change in physical activity*^d^
Men (n = 2487)	Remained employed	0.51 (0.45, 0.57)	0.60 (0.44, 0.76)	0.61 (0.45, 0.77)	0.63 (0.47, 0.79)
Entered retirement	0.41 (0.30, 0.51)	0.50 (0.32, 0.68)	0.50 (0.32, 0.68)	0.52 (0.34, 0.70)
Lost job^f^	0.56 (0.37, 0.76)	0.65 (0.41, 0.89)	0.66 (0.42, 0.90)	0.68 (0.43, 0.92)

Women (n = 2914)	Remained employed	0.44 (0.38, 0.50)	0.40 (0.23, 0.58)	0.41 (0.23, 0.58)	0.42 (0.24, 0.59)
Entered retirement	0.51 (0.39, 0.63)	0.47 (0.27, 0.67)	0.48 (0.28, 0.68)	0.48 (0.28, 0.68)
Lost job^f^	0.72 (0.56, 0.88)	0.68 (0.46, 0.91)	0.69 (0.46, 0.91)	0.69 (0.46, 0.92)

a *Model A* included age, educational attainment, and body weight at baseline as covariates.

b *Model B* included change in smoking status between baseline and follow-up.

c Change in dietary energy intake derived from the difference in total energy intake between baseline and follow-up estimated from food frequency questionnaires (FFQs).

d Change in physical activity based on transition between activity and inactivity levels derived from validated questionnaires.

e Mixed classification group was included in all models but not displayed.

f Includes individuals who left employment after baseline and either declared themselves unemployed at follow-up or did not indicate reason for being out of work.
