# Supplementary material for: Job-loss and weight gain in British adults: Evidence from two longitudinal studies
Source: Soc Sci Med. 2015 Oct;143:223–31. doi: 10.1016/j.socscimed.2015.08.052 (PMC4610948; doi:10.1016/j.socscimed.2015.08.052)
Supplement: Supplementary file 1 [file mmc1.docx]

**Figure S1:** Flow diagram for sample restriction in the European Prospective Investigation of Cancer (EPIC) Norfolk cohort and British Household Panel Survey (BHPS) for analyses reported in this study


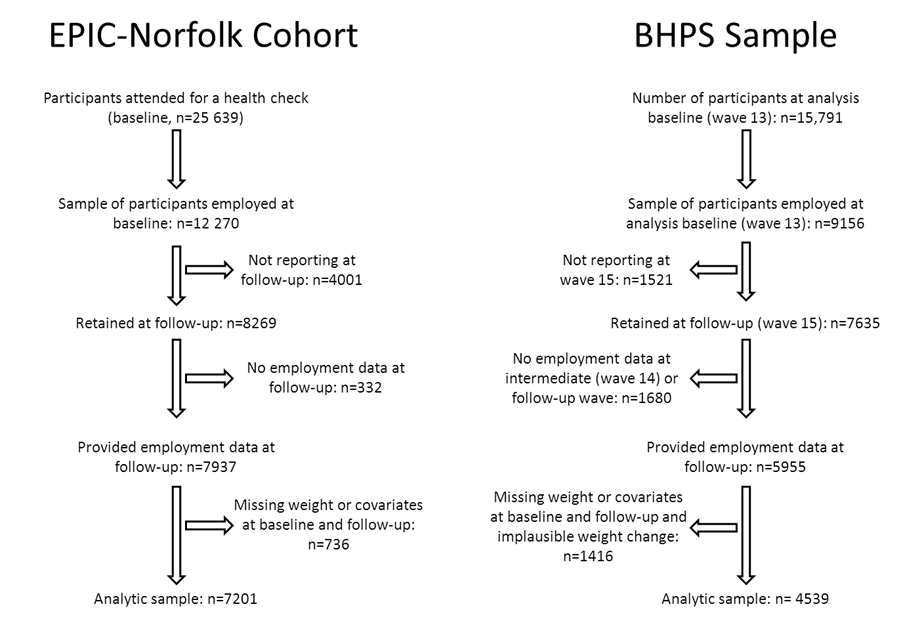


| **Table S1.** Comparison of analytic sample of employed adults with all employed adults in the EPIC-Norfolk cohort and the British Household Panel Survey**.** | | | | | |
| --- | --- | --- | --- | --- | --- |
|  | **EPIC cohort**  **(full baseline sample n=25 639)** | |  | **BHPS**  **(full baseline sample n=15 791)** | |
|  | **Analytic**  **Sample** | **All those employed at baseline** |  | **Analytic**  **Sample** | **All those employed at baseline** |
|  | *n=7201* | *n=12 270* |  | *n=4539* | *n=9156* |
| Women | 53% | 50% |  | 37% | 48% |
| Mean Age (s.d.) | 53.4 (6.5) | 53.1 (6.5) |  | 41.8 (11.6) | 40.2 (12.5) |
| Married | 86% | 85% |  | 62% | 57% |
| Education to A-level or degree | 62% | 61% |  | 50% | 50%^d^ |
| Higher social classes^a^ | 46%^b^ | 45%^c^ |  | 42% | 40%^e^ |
| Moderate/poor health | 12% | 14% |  | 20% | 21% |
| Current smoker | 10% | 13% |  | 24% | 26%^f^ |
| Mean BMI (s.d.) | 25.9 (3.6) | 26.1 (3.8) |  | 26.8 (4.3) | 25.9 (4.5) ^g^ |
| **^a^** Professional or managerial and technical professions; **^b^** Occupational social class in this sample have 53 missing cases;  **^c^** Occupational social class data missing in 106 cases; **^d^** For this sample, educational attainment missing in 782 cases;  **^e^** Occupational social class missing in 173 cases; **^f^** Current smoker status data missing in 665 cases; **^g^** Data for computing BMI missing in 1035 cases. | | | | | |

| **Table S2. Characteristics of the EPIC-Norfolk sample used in these analyses, by sex and overall, self-reported or measured at baseline.** | | | |
| --- | --- | --- | --- |
| **Descriptive characteristics** | **Women** (n=3784) | **Men** (n=3417) | **Overall** (n=7201) |
| **Age (mean years)** | 52.5 | 54.3 | 53.4 |
| **Marital status, num., (column %)** |  |  |  |
| Single | 122 (3.2%) | 119 (3.5%) | 241 (3.4%) |
| Married | 3090 (82.0%) | 3086 (90.7%) | 6194 (86.4%) |
| Widowed | 162 (4.3%) | 37 (1.1%) | 199 (2.8%) |
| Separated & Divorced  *(Missing 33/7201, 0.5%)* | 392 (10.4%) | 160 (4.7%) | 552 (7.7%) |
| **Education, num., (column %)** |  |  |  |
| No qualifications | 1116 (29.5%) | 776 (22.7%) | 1892 (26.3%) |
| O-level | 539 (14.2%) | 331 (9.7%) | 870 (12.1%) |
| A-level | 1529 (40.4%) | 1656 (48.5%) | 3185 (44.1%) |
| Degree | 600 (15.9%) | 654 (19.1%) | 1254 (17.4%) |
| **Social Class^a^, num., (column %)** |  |  |  |
| Unskilled | 135 (3.6%) | 85 (2.5%) | 220 (3.1%) |
| Partly skilled | 470 (12.5%) | 463 (13.7%) | 933 (13.1%) |
| Skilled occupations (manual) | 822 (21.9%) | 821 (24.2%) | 1643 (23.0%) |
| Skilled occupations (non-manual) | 670 (17.8%) | 384 (11.3%) | 1054 (14.7%) |
| Managerial and technical | 1416 (37.7%) | 1349 (39.8%) | 2765 (38.7%) |
| Professional  *(Missing 53/7201, 0.7%)* | 245 (6.5%) | 288 (8.5%) | 533 (7.5%) |
| **Smoking status, num.,** **(column %)** |  |  |  |
| Current smoker | 412 (10.9%) | 340 (10.0%) | 752 (10.4%) |
| Former smoker | 1145 (30.3%) | 1680 (49.2%) | 2825 (39.2%) |
| Never smoker | 2227 (58.9%) | 1397 (40.9%) | 3624 (50.3%) |
| **Self-rated health, num., (column %)** |  |  |  |
| Excellent | 780 (20.8%) | 728 (21.5%) | 1508 (21.2%) |
| Good | 2531 (67.5%) | 2231 (66.0%) | 4762 (66.8%) |
| Moderate | 424 (11.3%) | 412 (12.2%) | 836 (11.7%) |
| Poor  *(Missing 71/7201, 1.0 %)* | 13 (<1%) | 11 (<1%) | 24 (<1%) |
| **Body Mass Index (mean kg/m^2^)^b^** | 25.5 | 26.2 | 25.9 |
| **Body weight (mean kg)^b^** | 67.1 | 80.5 | 73.5 |
| **Annualised weight change (mean kg/year)^c^** | 0.50 | 0.48 | 0.49 |
|  |  |  |  |
| **^a^** Registrar General’s Social Class categories based on Occupation **^b^** Measured at baseline  **^c^** Difference between follow-up and baseline measured weight. | | | |

| **Table S3. Characteristics of the BHPS sample used in these analyses, by sex and overall, self-reported at baseline.** | | | |
| --- | --- | --- | --- |
| **Descriptive characteristics** | **Women** (n=1666) | **Men** (n=2873) | **Overall** (n=4539) |
| **Age (mean years)** | 42.0 | 41.6 | 41.8 |
| **Marital status, num., (column %)** |  |  |  |
| Single | 462 (27.7%) | 873 (30.4%) | 1335 (29.4%) |
| Married | 978 (58.7%) | 1826 (63.6%) | 2804 (61.8%) |
| Widowed | 37 (2.2%) | 20 (0.7%) | 57 (1.3%) |
| Separated & Divorced | 189 (11.3%) | 154 (5.4%) | 343 (7.6%) |
| **Education, num., (column %)** |  |  |  |
| No qualifications | 390 (23.4%) | 633 (22.0%) | 1023 (22.5%) |
| GCSEs, O-level | 513 (30.8%) | 731 (25.4%) | 1244 (27.4%) |
| A-level | 326 (19.6%) | 685 (23.8%) | 1011 (22.3%) |
| Degree | 437 (26.2%) | 824 (28.7%) | 1261 (27.8%) |
| **Social Class^a^, num., (column %)** |  |  |  |
| Unskilled | 61 (3.7%) | 87 (3.1%) | 148 (3.3%) |
| Partly skilled | 219 (13.3%) | 342 (12.0%) | 561 (12.5%) |
| Skilled occupations (manual) | 148 (9.0%) | 849 (30.0%) | 997 (22.2%) |
| Skilled occupations (non-manual) | 538 (32.7%) | 340 (12.0%) | 878 (19.6%) |
| Managerial and technical | 634 (38.5%) | 987 (34.7%) | 1621 (36.1%) |
| Professional | 46 (2.8%) | 231 (8.1%) | 277 (6.2%) |
| **Smoking status, num.,** **(column %)** |  |  |  |
| Current smoker | 399 (24.0%) | 668 (23.3%) | 1067 (23.5%) |
| Former smoker | n/a |  |  |
| Never smoker | n/a |  |  |
| **Self-rated health, num., (column %)** |  |  |  |
| Excellent | 431 (25.9%) | 853 (29.7%) | 1284 (28.3%) |
| Good | 870 (52.2%) | 1488 (51.8%) | 2358 (51.9%) |
| Fair | 283 (17.0%) | 439 (15.3%) | 722 (15.9%) |
| Poor and very poor | 81 (4.9%) | 93 (0.5%) | 174 (3.8%) |
| **Body Mass Index (mean kg/m^2^)** | 27.0 | 26.7 | 26.8 |
| **Body weight (mean kg)** | 72.5 | 84.4 | 80.0 |
| **Annualised weight change (mean kg/year)^b^** | 0.71 | 0.56 | 0.61 |
|  |  |  |  |
| **^a^** Registrar General’s Social Class categories based on Occupation except for eight men (0.3%) in armed forces (not shown); Missing 49/4539. **^b^** Difference between follow-up and baseline reported weights (approximately 24 months on average); Missing 3/4539. | | | |

| **Table S4.** Sensitivity analyses with data from the British Household Panel Survey. | | | |
| --- | --- | --- | --- |
| **Model** | | **Employment Transition** | Change and 95% CI in body weight (kg)  over follow-up period^a^ |
| **1** | Main analysis sample  (n=4539) | Remained employed | 0.60 (0.53, 0.68) |
|  |  | Entered retirement | 0.67 (0.09, 1.24) |
|  |  | Lost job | 1.56 (0.89, 2.23) |
|  |  |  |  |
| **2** | Exclude those with temp jobs (n=4319) | Remained employed | 0.61 (0.53, 0.68) |
|  |  | Entered retirement | 0.70 (0.10, 1.31) |
|  |  | Lost job | 1.73 (0.99, 2.46) |
|  |  |  |  |
| **3** | Exclude those not employed at wave prior to baseline (n=4222) | Remained employed | 0.59 (0.51, 0.66) |
|  |  | Entered retirement | 0.63 (0.05, 1.20) |
|  |  | Lost job | 1.39 (0.66, 2.13) |
|  |  |  |  |
| **4** | Exclude those not employed at 2 consecutive waves prior to baseline (n=3929) | Remained employed | 0.57 (0.49, 0.64) |
|  |  | Entered retirement | 0.72 (0.12, 1.32) |
|  |  | Lost job | 1.60 (0.81, 2.40) |
|  |  |  |  |
| **5** | Exclude those in poor health or substantial decline in health  (n=4101) | Remained employed | 0.59 (0.51, 0.66) |
|  |  | Entered retirement | 0.54 (-0.05, 1.13) |
|  |  | Lost job | 1.56 (0.84, 2.28) |
|  | |  |  |
| **^a^** Model included sex, age, educational attainment, smoking and weight at baseline as covariates. | | | |
